# Facing up to the wandering mind: Patterns of off-task laboratory thought are associated with stronger neural recruitment of right fusiform cortex while processing facial stimuli

**DOI:** 10.1016/j.neuroimage.2020.116765

**Published:** 2020-07-01

**Authors:** Nerissa Siu Ping Ho, Giulia Poerio, Delali Konu, Adam Turnbull, Mladen Sormaz, Robert Leech, Boris Bernhardt, Elizabeth Jefferies, Jonathan Smallwood

**Affiliations:** a Department of Psychology, University of York, England, UK; b Department of Psychology, University of Essex, England, UK; c Kings College London, London, UK; d McGill University, Montreal, Canada

**Keywords:** Off-task thought, Vivid detailed experience, Face processing, Fusiform cortex, Real-world neuroscience, Mind-wandering

## Abstract

Human cognition is not always tethered to events in the external world. Laboratory and real world experience sampling studies reveal that attention is often devoted to self-generated mental content rather than to events taking place in the immediate environment. Recent studies have begun to explicitly examine the consistency between states of off-task thought in the laboratory and in daily life, highlighting differences in the psychological correlates of these states across the two contexts. Our study used neuroimaging to further understand the generalizability of off-task thought across laboratory and daily life contexts. We examined (1) whether context (daily life versus laboratory) impacts on individuals’ off-task thought patterns and whether individual variations in these patterns are correlated across contexts; (2) whether neural correlates for the patterns of off-task thoughts in the laboratory show similarities with those thoughts in daily life, in particular, whether differences in cortical grey matter associated with detail and off-task thoughts in the para-hippocampus, identified in a prior study on laboratory thoughts, were apparent in real life thought patterns. We also measured neural responses to common real-world stimuli (faces and scenes) and examined how neural responses to these stimuli were related to experiences in the laboratory and in daily life - finding evidence of both similarities and differences. There were consistent patterns of off-task thoughts reported across the two contexts, and both patterns had a commensurate relationship with medial temporal lobe architecture. However, compared to real world off-task thoughts, those in the laboratory focused more on social content and showed a stronger correlation with neural activity when viewing faces compared to scenes. Overall our results show that off-task thought patterns have broad similarities in the laboratory and in daily life, and the apparent differences may be, in part, driven by the richer environmental context in the real world. More generally, our findings are broadly consistent with emerging evidence that shows off-task thoughts emerge through the prioritisation of information that has greater personal relevance than events in the here and now.

## Highlights

• Laboratory and daily life patterns of thought show both similarities and differences.
• Thoughts from both contexts have similar associations with medial temporal lobe grey matter.
• Laboratory off-task thoughts have stronger emphasis on content related to other people.
• Laboratory off-task thoughts are linked to greater neural activity when viewing faces.

## 1 Introduction

Human cognition is flexible and we often use imagination to focus on information pertinent to ourselves rather than events in the here and now. Often our minds wander to thoughts of a holiday, our family and friends, or to work related problems (Smallwood and Schooler, 2006, 2015). Experience sampling studies in both the laboratory and in daily life settings indicate we spend substantial amount of time engaging in patterns of thought that are, at best, loosely related to ongoing events in the external world (Kane et al., 2007; Killingsworth and Gilbert, 2010; Klinger, 1978). These self-generated thoughts often involve personally relevant topics, such as other people or other times and places (Poerio et al., 2015; Ruby et al., 2013; Smallwood, 2013; Smallwood et al., 2016). In the laboratory, studies suggest that self-generated experiences increase when the demands of the outside world decline (Smallwood et al., 2008; Teasdale et al., 1993), when we find the external environment uninteresting or lacking change (Giambra, 1993; Smallwood et al., 2009), or when we lack the motivation to engage with the task at hand (Seli et al., 2015). Recent evidence suggests that the capacity to self-generate patterns of thought unrelated to the external environment occurs through a process of prioritisation rooted in the neural architecture of the saliency network, including the dorso-lateral prefrontal cortex, and the anterior insula (Kam et al., 2019; Turnbull et al., 2019b).

Nevertheless, most existing research on self-generated thought relies on data gathered in laboratory settings with less meaningful semantic content, compared to the richness of the real world environment. An important question, therefore, is to what extent results derived from assessment of thoughts collected in the laboratory can be generalised to those in daily life. A small but growing number of studies have begun to explore and compare these two contexts, yielding evidence of both similarities and differences. For instance, in laboratory settings there is typically a robust negative association between working memory capacity and on-task thought during demanding tasks (e.g. McVay and Kane, 2009; Mrazek et al., 2012). However, the relationships with working memory and other executive functions are more complex when experience is sampled in daily life. In the real world, the link between executive abilities and off-task thoughts is less robust and varies as a function of concentration, that is, the ability to maintain attention to the ongoing task (Kane et al., 2007, 2017). More recently, Linz and colleagues (Linz et al., 2019) demonstrated that aspects of experiential content are correlated from laboratory and daily life (e.g. the degree of self-relevance or the focus on the future), whereas the degree of task focus was not. These studies, combined with theoretical accounts (Smallwood and Andrews-Hanna, 2013), emphasise that a more complete understanding of ongoing thoughts requires accounting for the broader context in which experience emerges. Our study leveraged the tools of cognitive neuroscience to further understand the similarities and differences between patterns of experiences observed in the laboratory and in the more complex context of daily life. This endeavour is important for developing ecologically valid neurocognitive accounts of self-generated thoughts, and also more generally for the emerging area of ‘Real-world Neuroscience’ (Hamilton and Huth, 2018; Matusz et al., 2019).

### 1.1 Study design

**Fig. 1:**
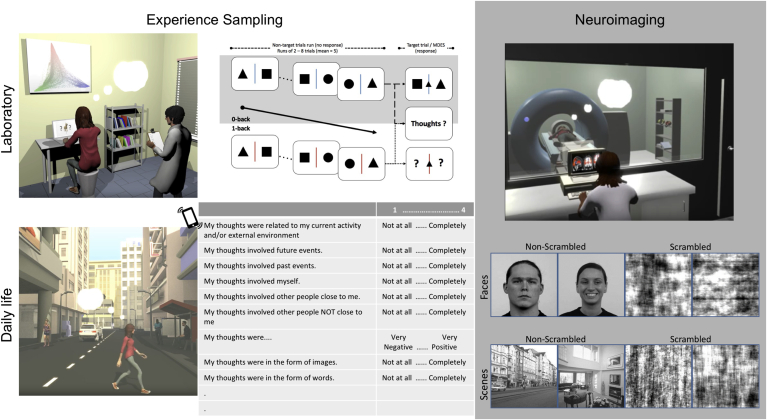
**Study design.** The study was designed to collect data from three different contexts: inside the MRI scanner, in the laboratory, and during daily life. **Left and middle panel (white background):** Experience sampling data was collected in two different contexts. (Top): In a laboratory context as participants performed a simple visual decision task with minimal semantic content. (Bottom): In a rich ecological context as participants went about their daily lives. In both contexts, participants described their ongoing experiences on multiple dimensions. **Right panel (grey background): S**tructural and functional neuroimaging data were collected while participants were lying in the scanner passively viewing faces and scenes.

Fig. 1 illustrates our study design. We sampled experiences across two situations: (i) in the laboratory while participants performed simple tasks in which they made spatial decisions about inanimate shapes (triangles, circles and squares) and (ii) in daily life where thoughts occurred in an ecologically valid context. We used a relatively large sample (*n* ​= ​77) recruited from a larger cohort of participants for whom we have previously documented thought patterns and associated neural correlates (for prior publications, see Ho et al., 2019; Turnbull et al., 2019a; Turnbull et al., 2019b; Wang et al., 2018b). In the same group of participants, we sought to establish patterns of experiences in the context of minimal content (the laboratory) and in the more ecological context of daily life. We first compared the patterns of experience obtained from these two contexts to quantify their similarities and differences. We also examined how individual variations in thought patterns from the real world and the laboratory are associated with measures of brain structure and function. In a previous study (Ho et al., 2019), we mapped patterns of laboratory ongoing thoughts to whole brain cortical thickness in a large cohort of individuals (those in the current study are a sub-sample of this cohort). We found that more detailed external thought patterns were associated with greater thickness in a region of the posterior hippocampus, whereas an adjacent anterior region was associated with more on-task thoughts. In the present study we examined whether these findings replicate with the ecologically valid thought patterns measured in the real world. Next, motivated by the importance of using meaningful stimuli in understanding cognition (Hamilton and Huth, 2018; Matusz et al., 2019), we examined whether individual differences in patterns of laboratory and daily life experiences were associated with neural responses to ecologically valid stimuli that are likely to be encountered outside laboratory contexts. Specifically, we measured neural activity while viewing faces and indoor or outdoor scenes. Scrambled versions of both sets of stimuli were included to act as controls.

## 2 Material and methods

### 2.1 Participants

Participants were healthy, right-handed, native English speakers, with normal or corrected-to-normal vision and no history of psychiatric or neurological illness. They were recruited as volunteers from the undergraduate and postgraduate student bodies at the University of York who were either paid or given course credits. The study was approved by the Department of Psychology and York Neuroimaging Centre, University of York ethics committees. All participants gave their written informed consent prior to taking part in any task or fMRI scanning, and were debriefed after completion of the study.

This study was a part of a larger project with a large cohort of individuals. Among these participants, seventy-seven (female ​= ​57; Age: Mean ​= ​19.66, SD ​= ​1.62, range ​= ​18–27) completed the experience sampling questionnaires in daily life. All but one of these participants, together with a bigger subgroup, completed the experience sampling questionnaires in the laboratory, totalling 199 participants (female ​= ​128; Age: Mean ​= ​20.11, SD ​= ​2.24, range ​= ​18–31). These data regarding experience sampling in the laboratory have been previously published with extensive descriptions of the neural correlates of different patterns of experience (see Ho et al., 2019; Turnbull et al., 2019a; Turnbull et al., 2019b; Wang et al., 2018b). For the purpose of comparison, we examined structural (cortical thickness) and functional (content localizer task) neuroimaging data for participants who had experience sampling data from both context. After excluding one participant with problematic structural brain data, valid brain data from seventy-three participants (55 females; Age: Mean ​= ​19.63, SD ​= ​1.65. Range ​= ​18–27) were used to quantify brain structural correlates of experience sampling in daily life. Only sixty-one participants successfully completed the functional neuroimaging task (content localizer task), three of whom were excluded because of missing data, leaving valid data for fifty-eight participants (female ​= ​43; Age: Mean ​= ​19.62, SD ​= ​1.69, range ​= ​18–27) for functional neuroimaging data analysis. Finally, we used resting state functional imaging data to embed any region identified from the content localizer analyses in a whole brain context, hence, we included all valid data for resting-state functional connectivity analysis. Of the initial 181 individuals who had scanned for resting state data, seven were excluded with missing or problematic data and another nine were excluded for excessive movement (see details in 2.5.2. Resting state fMRI under 2.5. Neuroimaging Data Preprocessing), leaving a total of 165 subjects (female ​= ​105; Age: Mean ​= ​20.18, SD ​= ​2.36, range ​= ​18–31). Please note that both the real world experience sampling data and the neural response to real world stimuli have not been previously published in any form. Due to time limitations we were not able to measure experience during the functional imaging session with passive viewing (content localizer task). Supplementary Table S1 provides a summary of the participant information for each of these groups.

### 2.2 Procedures

Laboratory experience sampling data were collected from participants who took part in a comprehensive set of behavioural assessments that captured different aspects of cognition, including the experiential assessment task, as well as other experimental tasks which were not analyzed in the current study. These tasks were completed over two to three sessions on different days, with the order of sessions counterbalanced across participants. Daily life experience sampling data were collected using participants’ own smartphones, which would alert them to respond to the experience sampling questionnaire five times a day for 7 ​days ​at quasi-random intervals as they went about their daily lives. Neuroimaging data was collected at the York Neuroimaging Centre.

### 2.3 Behavioural data

#### 2.3.1 Experiential assessment in the laboratory

The contents of ongoing thoughts in the laboratory were assessed using a 0-/1-back task, which were detailed in other studies from our laboratory (Sormaz et al., 2018; Turnbull et al., 2019a). Briefly, it is a simple visual decision making paradigm in which memory load is manipulated across alternate blocks of low-load (0-back) and high-load (1-back) conditions. At the end of each run of 2–8 (Mean ​= ​5) non-target trials, participants are required to respond to a target trial by deciding whether the probe shape (black-coloured circle, square or triangle) appears in the middle matches with the shape presented on the left or right hand side of the current (0-back) or previous (1-back) screen. In 20% of these target trials, instead of probes for shape, the “Thoughts ?” prompt appears and participants are then required to provide the subjective ratings of their ongoing thoughts on a four-point scale, from “Not at all” to “Completely”, for all 13 questions in the multidimensional experience sampling (MDES) questionnaire (see Table 1, rows marked as ‘L’ under column ‘Content’). Participants always rated their level of task focus first and then described their thoughts at the moment before the probe on a further 12 dimensions. These MDES probes occurred on a quasi-random basis to minimise the likelihood that participants could anticipate the occurrence of a probe. A similar approach has been used in our previous studies of on-going thoughts (e.g. Ruby et al., 2013; Smallwood et al., 2016).

**Table 1:** Multidimensional experience sampling questions.

Dimensions		Context ^[^a^]^	Questions	1	4
Task		D, L	My thoughts were related to my current activity and/or external environment.	Not at all	Completely
Future		D, L	My thoughts were about the future.	Not at all	Completely
Past		D, L	My thoughts were about the past.	Not at all	Completely
Self		D, L	My thoughts involved myself.	Not at all	Completely
Person ^[^b^]^		D	My thoughts involved other people close to me.	Not at all	Completely
		D	My thoughts involved other people NOT close to me	Not at all	Completely
Person		L	My thoughts involved other people.	Not at all	Completely
Emotion		D, L	My thoughts were ….	Very Negative	Very Positive
Images		D, L	My thoughts were in the form of images.	Not at all	Completely
Words		D, L	My thoughts were in the form of words.	Not at all	Completely
Vivid		D, L	My thoughts were vivid.	Not at all	Completely
Detailed		D, L	My thoughts were detailed and specific.	Not at all	Completely
Habit		D, L	My thoughts had recurrent themes similar to those that I have had before.	Not at all	Completely
Evolving		D, L	My thoughts tended to evolve in a series of steps.	Not at all	Completely
Deliberate		D, L	My thoughts were:	Completely Spontaneous	Completely Deliberate
NA ^[^c^]^		D	My thoughts were conflicting/interfering with what I am trying to achieve right now	Not at all	Completely
NA		D	My thoughts were helpful for goals that I am trying to achieve right now	Not at all	Completely
NA		D	My thoughts were helpful for goals that I am trying to achieve (or avoid) in the future	Not at all	Completely
NA		D	The content of my thoughts is important to me (i.e., it deals with something important in my life)	Not at all	Completely
NA		D	I was trying to control the progression of my thoughts	Not at all	Completely
NA		D	I wanted to have my thoughts	Not at all	Completely

Note:

For a full list of the PCA scores, see Supplementary Information Tables S2 and S3

a Denotes the setting in which question appeared: D ​= ​Daily Life; L ​= ​Laboratory.

b Average of the two items from daily life data entered as ‘Person’ dimension.

c NA = Not Available; these questions appeared in the Daily Life setting only, therefore, were not included in the PCA.

#### 2.3.2 Experiential assessment in daily life

Participants reported the content and form of their naturally occurring thoughts five times daily for seven days. This daily life experience sampling was administered using participants’ own smartphones in conjunction with SurveySignal Software (Hofmann and Patel, 2015). Participants received each signal as a text message with a link to an online survey created in Qualtrics. This survey required participants to consider the content of their thought immediately before answering the survey on various dimensions (see Table 1, rows marked as ‘D’ under column ‘Content’). We sought to assess the degree of similarity between the patterns of thought observed in the laboratory and daily life settings so we focused only on items that were presented in both contexts. We excluded 6 questions from the daily life questionnaire that had no analogue in the laboratory situation and averaged the ratings for the two questions related to the social content of thought that was present in the real world (MDES Dimension ​= ​‘Person’, see Table 1 Note ^[2]^). Signals occurred at random intervals between 09:00 and 21:00 with at least 30 ​min between adjacent signals. Participants were given two hours to answer the survey before the link expired, after that they were no longer able to provide a response (overall actual response rate ​= ​77.18%).

#### 2.3.3 Experiential assessment data analysis

To identify the internal structure of reports of ongoing thought content, principal components analysis (PCA) with varimax rotation was applied to the two MDES datasets using IBM SPSS Statistics (Version 25). Component scores were averaged for individual participants and transformed into z-scores, with outliers (>2.5) replaced with mean values (total outliers ​≤ ​2.44%).

Two analyses were adopted to assess the similarity of the PCA components from the laboratory and from daily life: (i) We conducted a single PCA which included thoughts in both contexts and calculated the correlations between an individual’s loadings in both situations and (ii) we calculated two separate PCAs in each context and the compared the similarity of the patterns produced using these independent analyses, as well as correlation of the component scores in the two contexts by individual participant. In the latter analysis, one of the components (emotion) showed similar relationships between items but the phase of these relationships was reversed in the two data sets (i.e. ‘Past’ was positive and ‘Emotion’ was negative in daily life, while ‘Emotion’ was positive and ‘Past’ was negative in the laboratory). Since PCA solutions are symmetrical, to facilitate interpretation, we reversed the scores in daily life for all of the analysis reported in the rest of this paper. Please note that the variation in patterns of experience across the 0-back and 1-back task have previously been described elsewhere (Turnbull et al., 2019a).

### 2.4 Neuroimaging data acquisition

Neuroimaging data were acquired at the York Neuroimaging Centre, York with a 3T GE HDx Excite MRI scanner using an eight-channel phased array head coil. First, a T1-weighted structural scan with 3D fast spoiled gradient echo (TR ​= ​7.8 ​s, TE ​= ​minimum full, flip angle ​= ​20°, matrix size ​= ​256 x 256, 176 slices, voxel size ​= ​1.13 x 1.13 ​× ​1 ​mm^3^) and a FLAIR sequence were acquired. Next, participants were asked to take a 9-min resting state fMRI (open eye) using single-shot 2D gradient-echo-planar imaging (TR ​= ​3 ​s, TE ​= ​minimum full, flip angle ​= ​90°, matrix size ​= ​64 x 64, 60 slices, voxel size ​= ​3 x 3 ​× ​3 ​mm^3^, 180 ​vol).

For some participants, this was followed by a content localizer task with single-shot 2D gradient-echo-planar imaging (TR ​= ​3 ​s, TE ​= ​minimum full, flip angle ​= ​90°, matrix size ​= ​64 x 64, 60 slices, voxel size ​= ​3 x 3 ​× ​3 ​mm^3^, 260 ​vol). Each participant, who had also participated in a semantic localizer task which has been analyzed previously (Zhang et al., 2019), completed one functional run (average running time: 8 ​min 35 ​s) for a single block of 312 trials. The task began with written instructions requesting the participants to passively view a series of stimuli presented at the centre of a grey screen (900 ​ms), each being separated by an inter-stimulus interval (ISI; 600 ​ms) when a small white fixation cross was displayed. At random intervals (10% trials), the fixation cross would change colour (white to green) and participants were required to press a button. This did not affect the presentation timing but was used to help participants maintain attentional focus.

**Fig. 2:**
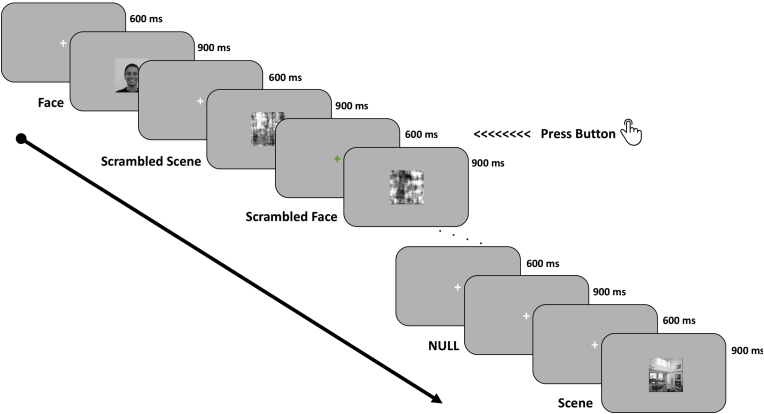
**Experimental paradigm of content localizer task.** While lying in the scanner, participants were requested to passively view a stream of face and scene images, in both non-scrambled and scrambled formats, randomly presented at the centre of the screen and separated by a fixation cross. In order to maintain participants’ attentional focus, they were required to press a button when the fixation cross appeared in a different colour, which would occur randomly for 10% of the trials.

The stimuli, presented in a quasi-random order, would either be a NULL stimulus (a normal fixation cross, 60 trials), or a grey-scale image (standardized in size of 256 x 256 pixel) of faces and scenes that are deemed to be commonly encountered in everyday life. Face images (non-scrambled ​= ​126, scrambled ​= ​42) were Caucasian adult faces selected from the Radboud Faces Database (Langner et al., 2010), with balanced number of positive (happy), negative (disgusted, sad and fearful) and neutral facial expression, gender (male vs female), as well as fixed proportion of gaze direction (frontal: left: right ​= ​3:2:2). Scene images (non-scrambled ​= ​42, scrambled ​= ​42) consisted of equal number of scenes related to city, indoor and nature (see Supplementary Information Fig. S2 for a sample of the experimental stimuli). Details of the paradigm is shown in Fig. 2.

### 2.5 Neuroimaging Data Preprocessing

#### 2.5.1 Cortical thickness

Cortical thickness is computed as the closest distance between the grey/white boundary and pial surface at each vertex across the entire cortex. This measure was derived from the T1-weighted images using models generated by FreeSurfer (Version 5.3.0; https://surfer.nmr.mgh.harvard.edu/) (Fischl, 2012) and preprocessed using the standard pipeline (for details, please refer to Dale et al., 1999; Fischl et al., 1999). Briefly, these steps include intensity normalization, removal of non-brain tissues, tissue classification, and surface extraction. We then visually inspected and, if necessary, manually corrected the cortical surface for each participant before registering surface data to an average spherical representation, fsaverage5, to improve the correspondence of locations across subjects. A surface-based smoothing with a 20 ​mm full-width-at-half-maximum (FWHM) Gaussian kernel was applied to reduce measurement noise without forgoing the capacity for anatomical localization (Lerch and Evans, 2005). Similar kernels have been used by our previous studies focussing on cortical morphology (Bernhardt et al., 2009, 2010, 2014; Valk et al., 2017).

#### 2.5.2 Resting state fMRI

Preprocessing steps for the MRI data were carried out using the SPM software package (Version 12.0) (http://www.fil.ion.ucl.ac.uk/spm/) based on the MATLAB platform (Version 16. a) (https://uk.mathworks.com/products/matlab.html). After removing the first three functional volumes to account for the magnetization equilibrium, the remaining data were corrected for motion using six degrees of freedom (x, y, z translations and rotations), and adjusted for differences in slice-time. Subsequently, the high-resolution structural image was co-registered to the mean functional image via rigid-body transformation, segmented into grey/white matter and cerebrospinal fluid probability maps, and were spatially normalized to the MNI space alongside all functional volumes using the segmented images and a priori templates (Ashburner and Friston, 2005). All the functional images were then smoothed using an 8 ​mm FWHM Gaussian kernel. We have also applied the MRI data denoising procedure to remove potential confounds of motion and other artefacts; and both of these steps were carried out using the Conn functional connectivity toolbox (Version 17. f) (https://www.nitrc.org/projects/conn) (Whitfield-Gabrieli and Nieto-Castanon, 2012). The denoising procedure was done by employing an extensive motion-correction procedure and denoising step, comparable to those reported in the literature (Ciric et al., 2017); and then entering the six realignment parameters and their second-order derivatives, a linear detrending term and the CompCor method that removed five principle components of the signal from white matter and cerebrospinal fluid (Behzadi et al., 2007) into linear model (Friston et al., 1996) as covariates of no interest. Volumes with excessive motion were also identified and scrubbed based on the conservative settings of motion greater than 0.5 ​mm and global signal changes larger than z ​= ​3 and participants who had more than 15% of their data affected by motion were excluded (Power et al., 2014). Finally, a band-pass filter between 0.009 ​Hz and 0.08 ​Hz was employed in order to focus on low frequency fluctuations (Fox et al., 2005).

#### 2.5.3 Task-based fMRI (content localizer)

Functional data were preprocessed and analyzed using a standard pipeline by FMRI Expert Analysis Tool (FEAT) from the FMRIB Software Library (FSL Version 6.0). Individual FLAIR and T1-weighted structural brain images were extracted using FSL’s Brain Extraction Tool (BET). Structural images were registered to the MNI152 template using FMRIB’s Linear Image Registration Tool (FLIRT). Spatial preprocessing of the functional data included motion correction via MCFLIRT, slice-timing correction with Fourier space time-series phase-shifting, and spatial smoothing using a Gaussian kernel of FWHM 6 ​mm. A high-pass temporal filtering (sigma ​= ​100 ​s) was also applied in order to remove temporal signal drift and participants with excessive motions were excluded.

### 2.6 Neuroimaging data analysis

#### 2.6.1 Cortical thickness

Cortical thickness data were analyzed using SurfStat for matlab [http://www.math.mcgill.ca/keith/surfstat/] (Worsley et al., 2009). One of our recent studies revealed that cortical thickness of two adjacent areas at the medial temporal lobe (MTL) were associated with individual variations of detailed and off-task thoughts based on PCAs derived from laboratory experience sampling data (Ho et al., 2019). This indicated that patterns of ongoing thought might be related to stable features of brain anatomy. To extend this investigation regarding the association of stable neural features to patterns of ongoing thoughts in daily life, we extracted cortical thickness from clusters in our previous study, and used an ANOVA with repeated measures (Greenhouse-Geisser corrected) to test whether cortical thickness of these brain regions would be associated with patterns of ongoing thought in daily life. Consistent with our prior study, we included both age and gender as covariates of no-interest.

#### 2.6.2 Resting state fMRI

We used the resting state data to embed any region identified through the content localizer analyses in a whole brain context. Seed-based functional connectivity analyses were conducted based on the binarised seed ROIs that were highlighted through the content localizer task. Individual connectivity map for each participant were obtained by extracting the average BOLD signal within the time series of the ROI and then correlated with the time course of each individual voxel in the rest of the brain. These correlation results were then transformed to Fisher’s Z-scores to produce the standardized functional connectivity. Group-level inferences on the connectivity map were made based on one-sample t-tests, thresholded at Z ​= ​3.1 (Eklund et al., 2016). The resulting brain networks were visualized using Connectome Workbench (v1.3.2). [https://www.humanconnectome.org/software/connectome-workbench].

#### 2.6.3 Task-based fMRI (content localizer)

This analysis included a total of 58 participants for whom there were valid localizer data, as well as measures of experience in both the laboratory and daily life. For our first-level analysis, we included four explanatory variables namely, faces, scrambled faces, scenes, scrambled scenes, each modelled for the time period of 900 ​ms during which the respective type of image was presented. This was convolved with the hemodynamic response function and contrasts were created to assess brain activity related to main effects of faces (faces ​> ​scrambled faces) and scenes (scenes ​> ​scrambled scenes), as well as comparisons of these two types of stimuli ((faces ​> ​scrambled faces) ​> ​(scenes ​> ​scrambled scenes) and vice versa). Please note that we assessed faces with different emotional expressions (positive, neutral and negative). These were modelled separately and analysis indicated that there were no effects specific to a particular facial expression, accordingly we do not report these analyses in the main body of the text.

Group-level analysis was modelled by entering the PCA parameters, that is, averages (mean PCA scores from daily life and laboratory) and differences (PCA scores from daily life minus scores from laboratory) for all relevant PCAs (see 2.6.1 Experience sampling analysis), into a linear model as explanatory variables for predicting neural activity represented by the contrast maps generated from the first-level modelling. All spatial maps were thresholded at Z ​= ​3.1 (Eklund et al., 2016). The resulting brain networks were visualized using Connectome Workbench (v1.3.2). [https://www.humanconnectome.org/software/connectome-workbench].

### 2.7 NeuroSynth reverse inference (meta-analysis)

Automated meta-analyses were conducted using the NeuroSynth decoder (http://neurosynth.org/decode; Yarkoni et al., 2011) to make quantitative inferences on the results identified from fMRI analysis. Unthresholded connectivity maps obtained from group analysis were submitted to NeuroSynth, which then computed the spatial correlations between each of these maps and a large database of meta-analytic maps (*n* ​= ​11,406) for different cognitive terms (e.g., autobiographic, memory, task, emotional regulation). The top 15 terms (singular and plural forms of the same term were combined), excluding neuroanatomical terms, exhibiting the highest positive correlation with each unthresholded map submitted were extracted and presented as word clouds. Size and colour of the font reflects the strength of the relationship. This analysis allows us to quantify the reverse inferences that would be drawn from the functional maps by the larger neuroimaging community.

## 3 Results

### 3.1 Experiential assessment

**Fig. 3:**
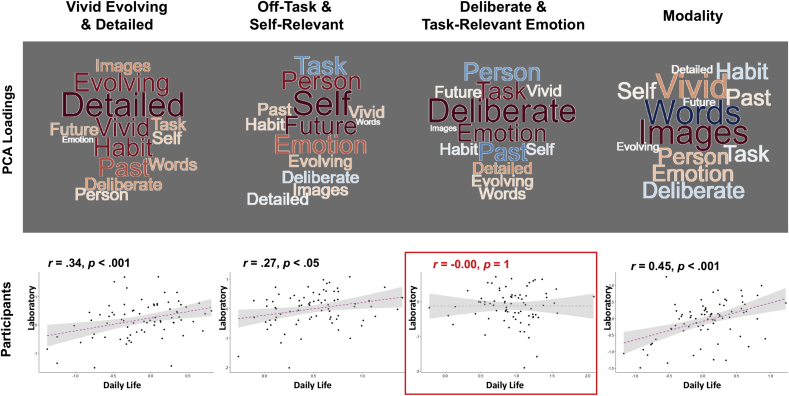
**Combined analysis of MDES questionnaires obtained from both laboratory and daily life in a single PCA**. Word clouds (Top) highlighting the components loadings from both contexts, with the font size representing the strength of the relationship, and colour indicating the direction of the relationship (warm colours ​= ​positive and cooler colours ​= ​negative). Correlations between daily life and laboratory results for each PCA component calculated by individual participant’s component scores by context (Bottom).

Our first analysis combined MDES results obtained from both contexts and decomposed these in a single PCA. (see Supplementary Information Fig. S2 for the scree plot). The decompositions can be summarized along four dimensions: (i) ‘Vivid Evolving and Detailed’ - describing patterns of thought with the highest loadings on ‘Detailed’ and ‘Evolving’, (ii) ‘Off-Task and Self-Relevant’ with low loadings on ‘Task’ and higher loadings on ‘Self’, (iii) ‘Deliberate and Task-relevant Positive Emotion’ and (iv) ‘Modality’ with high loadings on ‘Images’ and low loadings on ‘Words’. Word clouds representing loadings for each component is shown in Fig. 3 (top panel). To assess how consistent these were across the two contexts, we summarized each individual’s PCA scores by the laboratory and daily life situations and correlated these values. We found that all components, except the ‘Deliberate and Task-relevant Positive Emotion’ component were correlated across the two situations (Fig. 3, bottom panel). Inspection into the summary statistics of the individual MDSE questions (see Supplementary Information Table S4) showed that there were relatively larger variabilities of “Emotion” and “Deliberate” thoughts, which may be the driving cause of this non-correlation of the third component.

Next we examined the PCA structure independently across the two situations. For consistency across contexts we calculated four components in both cases (see Supplementary Information Fig. S2 for the scree plots) which explained 59.83% and 54.85% of the total variance in ongoing thought contents in the laboratory and in daily life respectively.

**Fig. 4:**
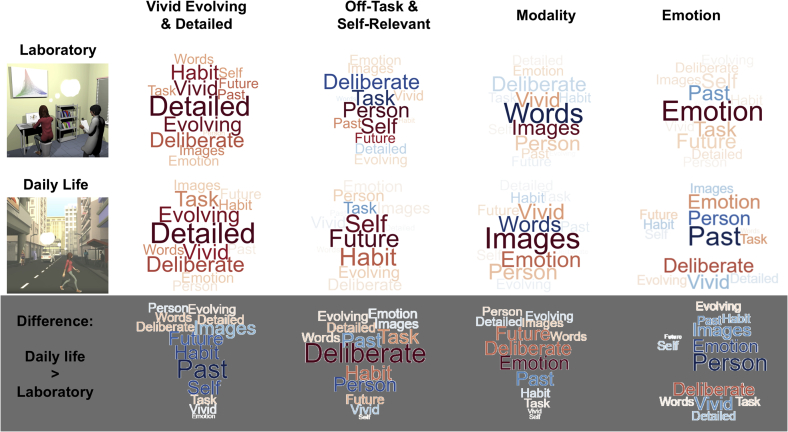
Independent analyses of MDES questionnaires obtained from laboratory and daily life with separate PCAs. Word clouds illustrating loadings of the 13 multidimensional experience sampling (MDES) questions on each of the four PCAs derived from data collected in laboratory (Top) and in daily life settings (Middle), as well as the difference between these settings (Bottom: daily life minus laboratory loading). Font size in these word clouds highlight the strength of the relationship, and colour indicates the direction of the relationship (warm colours ​= ​positive and cooler colours ​= ​negative).

Representation of the four components by word clouds (Fig. 4) revealed that independent decompositions of the datasets from these two settings showed broadly similar internal structures of ongoing thought patterns, which are also broadly consistent with the structure revealed by the combined analysis using single PCA. These four dimensions are: (i) ‘Vivid Evolving and Detailed’ (ii) ‘Off-Task and Self-Relevant’ (iii) ‘Modality’ and (iv) ‘Emotion’ with opposite loadings on ‘Emotion’ and ‘Past’ (i.e. positive loadings emotion and negative loadings on the past). Compared to daily life thought patterns, ‘Vivid Evolving and Detailed’ thoughts in the laboratory were more self-focused and recurrent, and tended to be past or future oriented; whereas ‘Off-Task and Self-Relevant’ laboratory thoughts were more focused on other people and the past, but were less deliberate and recurrent (see bottom panel of Fig. 4 for further comparisons).

**Fig. 5:**
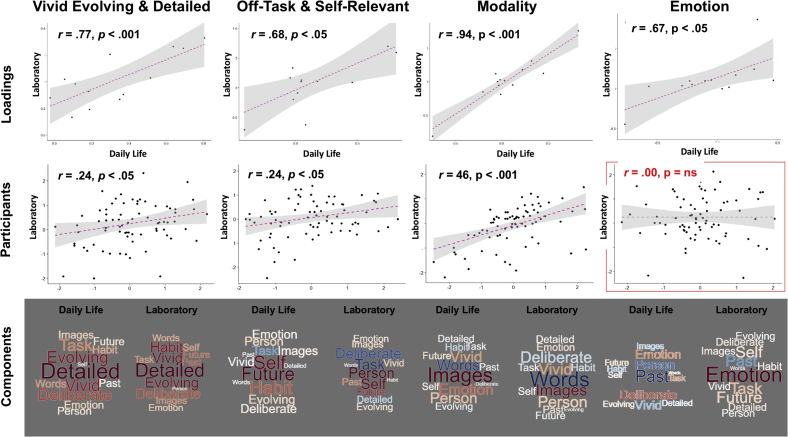
**Relationships between thought patterns in laboratory and daily life**. Correlations between daily life and laboratory results for each PCA component calculated by (i) component loadings of the 13 experience sampling questions grouped across the entire sample (Top), and (ii) individual participant’s component score (Middle). Word clouds (Bottom) highlighting the components loadings from both contexts, with the font size representing the strength of the relationship, and colour indicating the direction of the relationship (warm colours ​= ​positive and cooler colours ​= ​negative).

To quantify the relationship between thought patterns recorded across the two contexts, we correlated both PCA loadings and individual PCA scores obtained from both contexts. Significant positive correlations were observed for PCA loadings (internal structure of thought contents summarized by group ratings in the two settings) of all four components (Fig. 5, top panel), and moderate correlations were found for individual PCA scores (individual participants’ actual thought component ratings across the two settings) in three of these four components (‘Vivid Evolving & Detailed’, ‘Off-Task & Self-Relevant’, ‘Modality’ but not ‘Emotion’) (Fig. 5, middle panel). These results suggested that ongoing thought contents across contexts were broadly consistent in structure (PCA loadings) and were correlated across participants. Similar to the combined analysis, the component showing the strongest loading for emotion was the least well correlated across contexts.

Having established both similarities and differences in the patterns of ongoing thoughts in the laboratory and in daily life, we next examined their neural correlates using the results from independent analyses (as they are not constrained by the assumption that thoughts in the two contexts are having the same internal structure). Eight parameters were derived from the independent PCA results: four averages of the PCA loadings across contexts and four differences for daily life minus laboratory PCA loadings. These parameters were transformed into z-scores, with outlying values (>2.5) replaced by the mean (overall percentage of outliers ​< ​3.23%). They were then used to examine the neural associations with patterns of ongoing thoughts and both anatomical and functional indices of brain organisation.

### 3.2 Cortical thickness

**Fig. 6:**
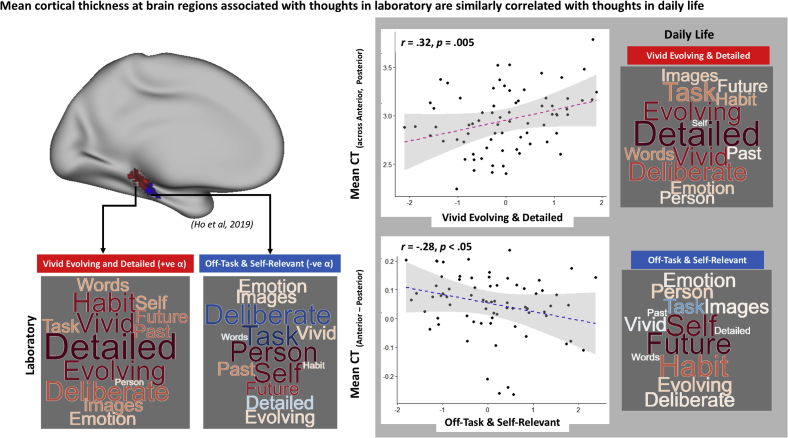
**Mean cortical thickness of para-hippocampus related to patterns of thought in the laboratory is also associated with thoughts in daily life. Left panel (white background):** Locations of the two left para-hippocampal regions of interest found to be associated with Detailed and On-Task thoughts in (Ho et al., 2019) (Top). Word clouds highlighting the thought content loadings in laboratory (Bottom). **Right panel (grey background):** Scatterplot showing that mean cortical thickness across both regions in para-hippocampus is associated with detailed thoughts in daily life, with the word cloud representing loadings of the relevant thought content (Top). Scatterplot showing greater mean cortical thickness at anterior compared to posterior region in the para-hippocampus is associated with off-task thoughts in daily life, with the word cloud representing loadings of the relevant thought content (Bottom). **Note:** For all word clouds, font size represents the strength of the relationship and colour indicates the direction of the relationship (warm colours ​= ​positive and cooler colours ​= ​negative).

Our cortical thickness analysis examined whether there are similarities between the structural correlates associated with patterns of thought in the laboratory and those observed in daily life. To achieve this, mean cortical thickness was extracted from the left medial temporal regions associated with ‘Vivid Evolving & Detailed’ thought and ‘On-Task’ (negatively associated with ‘Off-task & Self-relevant’) thoughts from the larger laboratory sample reported in our prior study (Ho et al., 2019). We then ran a repeated measures ANOVA in which the two averages of the cortical thickness in these two regions of the para-hippocampus were dependent variables, while the two average loadings on Detailed and Off-task components from the real-world experience sampling were explanatory variables. Consistent with our prior study, we included both age and gender as covariates of no-interest. We found two patterns of associations between cortical thickness and ongoing experience. First, there was a main effect of the Detailed component (*F*(1, 68) ​= ​8.01, *p* ​< ​.01) which reflected a positive correlation between Detailed thoughts in daily life and cortical thickness across both regions of the para-hippocampus (*r* ​= ​0.32, *p* ​= ​.005). In addition, a region by Off-task interaction (*F* (1, 68) ​= ​7.88, *p* ​< ​.01) indicated that a greater on-task focus was linked to greater thickness in the anterior relative to the posterior region of the left para-hippocampus (*r* ​= ​−0.28, *p* ​< ​.05) (Fig. 6).

Together these patterns are broadly consistent with our previous findings using laboratory based thought sampling (Ho et al., 2019). They indicate that task focused thoughts are more closely linked to greater thickness in the anterior part of the left para-hippocampus, whereas detailed thoughts are more closely associated with the posterior region. These analyses, therefore, have established a broad similarity in the ‘brain structure – experience’ relationship, regardless of whether thoughts were sampled in the laboratory or in the more ecologically valid context of one’s daily life.

### 3.3 Face and scene processing related neural activity

**Fig. 7:**
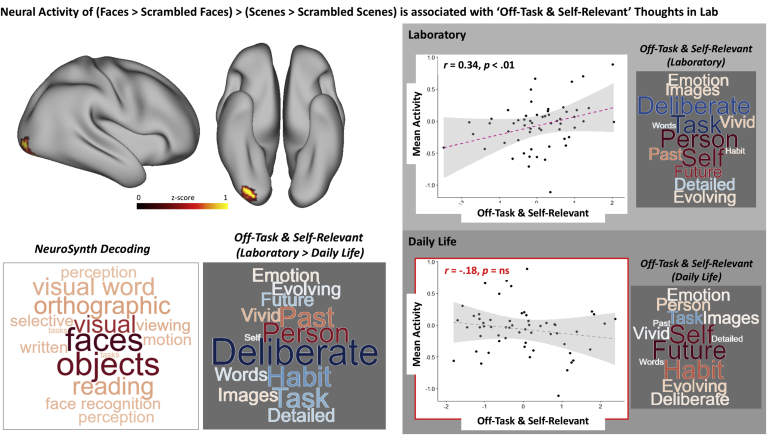
**Neural activity during face and scene processing is related to Off-Task thoughts in laboratory. Left panel (white background):** Cluster in a region of right fusiform cortex has neural activity specific to processing faces that is significantly correlated with ‘Off-task & Self-relevant’ thoughts in the laboratory, more than in daily life (Z ​= ​3.1) (Top). Word clouds on the left and right show the decoding of this region using NeuroSynth (Yarkoni et al., 2011) and Loadings of (Laboratory ​> ​Daily Life) for ‘Off-task & Self-relevant’ thought contents respectively (Bottom). **Right Panel (grey background):** Scatterplots showing the association between neural activity extracted from the cluster and ‘Off-task & Self-relevant’ thoughts in the Laboratory (Top) and in Daily Life (Bottom), with word clouds showing the loadings of the relevant thought contents. **Note:** For all word clouds, font size represents the strength of the relationship, and colour indicates the direction of the relationship (warm colours ​= ​positive and cooler colours ​= ​negative).

Next, to understand whether patterns of ongoing experience are related to the neural responses to faces and scenes, we analyzed the neural activity using a generalised linear model (GLM). First, we formulated a high level contrast (Faces ​> ​Scrambled Faces) ​> ​(Scenes ​> ​Scrambled Scenes) to identify neural activity specific to either type of content. We then regressed this contrast, within a single linear model, against (i) overall levels of experience for both situations, as well as (ii) differences in these experiences across the two contexts. We found a significant cluster that showed a pattern of activity consistent with an interaction between stimulus type and the difference in off-task thoughts across contexts. Greater neural activity was observed in a cluster near the right fusiform gyrus (including the right inferior and superior lateral occipital cortex, occipital pole and fusiform gyrus) when viewing faces compared to viewing scenes. Neural activity in this region increased with levels of ‘Off-task & Self-relevant’ thoughts in the laboratory, but not for those in daily life (shown by scatterplots in Fig. 7). Decoding this region using NeuroSynth confirmed a pattern of neural responses seen for faces and other objects (see left hand word cloud). Together these results show that individuals who tended to have more ‘Off-task & Self-relevant’ thought in the laboratory also tended to show greater activity in a region of right fusiform cortex when viewing faces compared to scenes. Importantly, an apparent bias linked to social information is also reflected in the loadings of off-task thoughts, that is, more thoughts on ‘Person’ are associated with off-task thoughts in the laboratory than in daily life (summarized in the word clouds on the right panel in Fig. 7.)

**Fig. 8:**
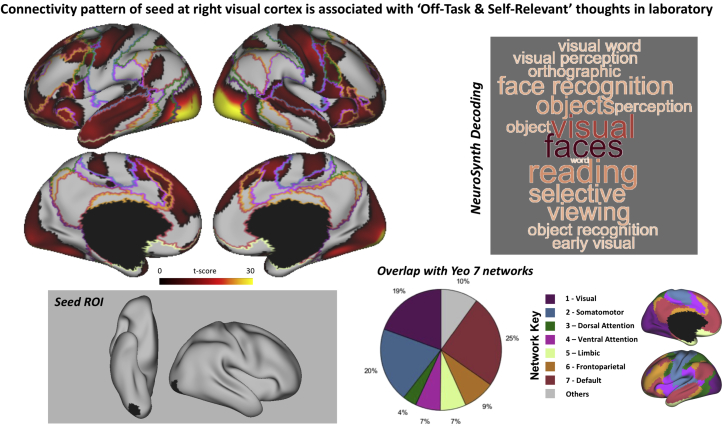
The functional network involving the region of fusiform cortex exhibiting neural activity associated with off-task thought in the laboratory. The spatial map (Top-left) was generated using a seed-based connectivity in which the seed was the region identified by our prior analyses (Z ​= ​3.1) (Bottom-Left). The word cloud shows the decoding of this region using NeuroSynth (Yarkoni et al., 2011) with font size representing the strength of the relationship (Top-right). The pie chart (Bottom-right) highlights the percentage of voxels within each connectivity map that fall within each of the seven large scale networks identified by Yeo et al. (2011). The colour scheme used in the pie chart shows the correspondence to Yeo Seven network parcellation which is presented in the Network Key.

Finally, to embed the region of right fusiform cortex in a whole brain functional context, we conducted a seed-based resting state functional connectivity analysis using the region of interest at the right fusiform cortex (see Methods). We identified a network of regions that had a similar time course. These included both unimodal regions (i.e. visual and motor cortex), and transmodal regions including lateral temporal cortex, anterior insula and the tempo parietal junction. Decoding of this spatial map using Neurosynth identified terms confirming that this pattern of neural activity is often seen when individuals actively engage with the external environment (terms like “visual”, “faces”, “objects”, “reading”) (Fig. 8).

## 4 Discussion

We leveraged the tools of neuroimaging to gain a better understanding of differences and similarities between patterns of ongoing thoughts across laboratory and naturalistic real world contexts. Application of principal components analysis (PCA) to experience sampling data obtained from both laboratory and daily life contexts revealed similar patterns of personal, temporal and social thought content during off-task states. We also found similarities between the architecture of the medial temporal lobe and patterns of thought in the laboratory and in daily life. Extending recent work by Linz et al. (2019), these findings suggest that laboratory-based measures provide a reasonable approximation for real world patterns of thought. However, we also found important differences across contexts: laboratory off-task thoughts were focused to a greater degree on other people compared to thoughts sampled in real world settings. This difference in experiential content across context was mirrored at a neural level: individuals who tended to experience more off-task thoughts in the laboratory showed heighted activity in a region of right fusiform cortex when processing external social information (viewing faces).

From a methodological viewpoint, our study provides insight into the generalizability between cognition, as measured in the controlled context of the laboratory, and in the more contextually rich real world (Hamilton and Huth, 2018; Matusz et al., 2019). We found broad similarities between the patterns of experiences across contexts in terms of: (i) their patterns of loadings, (ii) their variations across individuals and (iii) their associations with grey matter structures in the para-hippocampus. Given the role of the medial temporal lobe in memory, this latter finding provides initial evidence that memory systems play a vital role in driving patterns of imaginative experience in the real world as it is assumed to play in laboratory settings (Corballis, 2015).

Despite broad similarities, we also identified differences across contexts. Notably, off-task laboratory thoughts were more social and occurred more spontaneously than those experienced in the real world. These differences were reflected in the functional brain activity patterns such that individuals who experienced more off-task thoughts in the laboratory responded more strongly to similarly social *external* stimuli (faces); while similar relationship was not observed for real world off-task thoughts. Our findings add to the small but growing body of evidence highlighting how differences between off-task thoughts across contexts relate to other aspects of cognition such as attentional control (Kane et al., 2017). Compared to the highly controlled conditions of a laboratory task, daily life provides people with a much richer and engaging environment within which their ongoing thoughts unfold. We also found differences in the coupling between positive emotion and the degree of intention in on-going experience between the two settings, with daily life thinking characterised by positive deliberate thought more than in the laboratory. We speculate that this may occur because the laboratory is relatively devoid of opportunities to engage in activities that participants enjoy performing. This finding provides a novel insight into evidence that motivation is especially important for the experience of off-task thoughts under laboratory conditions (Seli et al., 2015, 2016, 2018). It is possible, therefore, that in the real world individuals often engage in tasks that they enjoy performing, which are often lacking from laboratory situations, and so may explain why single questions regarding task focus does not generalise across the two situations (Linz et al., 2019).

Although there are differences in thought patterns assessed across laboratory and daily life contexts, our analysis does not preclude the study of ongoing experience under controlled settings. Instead, our study highlights that generalising from laboratory findings to daily life should be done cautiously, and ideally, future studies would assess experiences across both contexts. Laboratory measures of experience, especially using techniques such as MRI, provide a greater degree of precision when investigating underlying cognitive mechanisms, something that can be enhanced by their integration with sampling in ecological real world situations. We have shown here that it is possible to combine the detailed measures of cognitive neuroscience, with the richer, more naturalistic measurements obtained in daily life in a way that can help constrain theoretical accounts of ecologically valid aspects of cognition (Hamilton and Huth, 2018; Matusz et al., 2019). A useful approach, therefore, would be to combine task-based neuroimaging studies with experience-sampling studies that measure naturally occurring experience outside the laboratory. This would allow the identification of the specific patterns of thought that are most meaningful in the real world. Moreover, in combination with neuroimaging work, it would set the foundations for an integrative approach in which these everyday thought patterns would simultaneously enhance the ecological validity of neuroimaging work and provide a mechanistic and neural explanation for whether, when, and how on-going experience would help or hinder navigation of the social world.

While our study used neuroimaging to provide evidence of both similarities and differences in the patterns of thought observed in the laboratory and in daily life, it nonetheless leaves open several important questions. One such issue concerns the mechanism that underpins the observed association between elevated neural responses to faces and off-task thought in the laboratory. Our data show a neurocognitive association across individuals between laboratory thoughts and visual processing of faces, and there are several different mechanisms that could explain this. One possibility is that it could reflect a process through which social information attracts attention, perhaps at an early perceptual level. Our prior studies have highlighted that patterns of thought are prioritised across tasks in a contextually appropriate manner by regions of the ventral attention or saliency network (as defined by Yeo et al., 2011), and in particular the dorso-lateral prefrontal cortex (Turnbull et al., 2019a, 2019b). This neural system is important for prioritising different types of relevant internal and external stimuli (Seeley et al., 2007), which may, in part, enabled by this network’s ability to causally influence other large scale networks (Bonnelle et al., 2012; Goulden et al., 2014). Alternatively, studies have shown that regions of fusiform cortex are activated during imagination of the future and the past (Addis et al., 2007; St. Jacques et al., 2008). An alternative implication of our data, therefore, is that individuals who prioritise thoughts about other people in the laboratory possess more elaborate social representations in memory, which in turn could lead to the greater neural response when viewing faces observed in our study. More generally, we speculate that the implementation of motivationally salient information in self-generated thinking, may be influenced by the saliency network’s ability to prioritise goal relevant information, a question that will be an important focus for future work.

It is important to note that our observation that off-task thought in the laboratory and neural activation while viewing faces are associated should not be seen as an alternative to accounts of off-task thought which emphasise the need to maintain attention on external tasks. For example, there is a large body of evidence demonstrating that off-task thought is related to an inability to constrain attention to external task-relevant information. This perspective is often referred to as the executive-failure view (McVay and Kane, 2010, 2012; Robison and Unsworth, 2017, 2018) (see Randall et al., 2014 for a meta-analysis review) and is supported by a growing body of neurocognitive evidence (Turnbull et al., 2019a, 2019b; Wang et al., 2018a). Rather than framing these accounts as being in competition, the heterogeneous nature of on-going thought encourages us to consider them as complementary accounts which describe different features of the complex landscape of experience (see also Christoff et al., 2016). In this context, our study is important because it highlights that constructs such as ‘off-task thought’ can share broad similarities at both self-report and neural levels, as well as important contextual differences. Moving forward, it should be a priority for future work to focus on understanding how different features of our neurocognitive architecture influence experience in different contexts, and how they are implicated in different types of self-generated content (Smallwood and Andrews-Hanna, 2013). For example, in the context of face processing, Denkova et al. (2018) found that off-task thought led to an attenuation of face relevant event related components in the scalp electroencephalogram (including the n170). It would be informative in the future to compare both neural and experience sampling data in laboratory contexts using tasks with more ecological content to understand in greater detail whether the neural correlates of momentary changes in attention vary across different contexts.

We close by considering the broader implications of our results for accounts of off-task thought, particularly with respect to the role they play in social life (Poerio and Smallwood, 2016). Contemporary neuroscientific enquiry has documented the breadth of neural regions engaged during social cognition (Bzdok et al., 2016; Mars et al., 2012), observations that confirm the importance of social cognition from a neuro-scientific perspective (Schilbach et al., 2008). Likewise, prior experience sampling and other psychological work has shown that patterns of ongoing thought are often social (e.g. Ruby et al., 2013) and can have important implications for health and well-being (Vatansever et al., 2019). For example, positive thoughts about other people can improve mood (Poerio et al., 2015), reduce loneliness (Poerio et al., 2016a), promote social and emotional adjustment (Poerio et al., 2016b), and facilitate personal goal development (Medea et al., 2018). Overall, the prevalence of social information during self-generated thought, and its ability to predict success and failure in the social world, is consistent with the idea that we use ongoing thoughts to focus on personally important information and contextually relevant goals, a view known as the *current concerns* account (Klinger, 1978; Klinger and Cox, 1987). Our results are broadly in line with this perspective because they suggest that individuals who prioritise imagining socially relevant information during a simple laboratory task show heightened processing of faces in selective face processing regions. Given the importance of social processing to our species, a more thorough neuro-cognitive account of how individuals use their imagination in daily life may help us to understand the conditions under which we can survive and thrive in the complex social landscape of daily life.

## CRediT authorship contribution statement

**Nerissa Siu Ping Ho:** Conceptualization, Methodology, Validation, Formal analysis, Writing - original draft, Writing - review & editing, Visualization. **Giulia Poerio:** Conceptualization, Methodology, Investigation, Writing - review & editing. **Delali Konu:** Investigation. **Adam Turnbull:** Investigation. **Mladen Sormaz:** Methodology, Investigation. **Robert Leech:** Resources. **Boris Bernhardt:** Methodology, Writing - review & editing. **Elizabeth Jefferies:** Writing - review & editing. **Jonathan Smallwood:** Conceptualization, Methodology, Writing - review & editing, Supervision, Funding acquisition.
