# Supplementary material for: Facing up to the wandering mind: Patterns of off-task laboratory thought are associated with stronger neural recruitment of right fusiform cortex while processing facial stimuli
Source: Neuroimage. 2020 Jul 1;214:116765. doi: 10.1016/j.neuroimage.2020.116765 (PMC7284321; doi:10.1016/j.neuroimage.2020.116765)
Supplement: Multimedia component 1 [file mmc1.docx]

**Supplementary Information**

Table S1: Participant information summary

|  | MDES (PCA) | Cortical Thickness | Content Localizer | Resting-state |
| --- | --- | --- | --- | --- |
| DL only | 1 | Not relevant | Not relevant | 0 |
| Lab only | 123 | Not relevant | Not relevant | 109 |
| DL & Lab | 76 | 74 | 61 | 56 |
| Exclusion | None | 1 (Problematic) | 3 (Missing) | 7 (Problematic)  9 (Movement) |
| Total Included | DL = 77  Lab = 199 | 73 (DL & Lab) | 58 (DL & Lab) | Lab = 165 |
| Overlap with  Ho et. al. | First reported (DL)  181 (Lab) | 65 | First reported | 165 |

Notes:

DL = Daily Life, Lab = Laboratory

**Table S2**: PCA scores of the multidimensional experience sampling questions in the laboratory

| Questions | Detail | Off-Task | Modality | Emotion |
| --- | --- | --- | --- | --- |
| Task | 0.31 | -0.71 | -0.05 | 0.24 |
| Future | 0.38 | 0.6 | -0.05 | 0.17 |
| Past | 0.42 | 0.34 | 0.12 | -0.44 |
| Self | 0.28 | 0.7 | 0.02 | 0.11 |
| Person | 0.13 | 0.76 | 0.19 | 0.13 |
| Emotion | 0.19 | 0.1 | 0.13 | 0.86 |
| Images | 0.33 | 0.13 | 0.76 | 0.1 |
| Words | 0.27 | -0.09 | -0.82 | 0 |
| Vivid | 0.67 | 0.17 | 0.31 | 0.04 |
| Detailed | 0.73 | -0.2 | 0.03 | 0.1 |
| Habit | 0.61 | 0.09 | -0.15 | 0.05 |
| Evolving | 0.65 | 0.11 | 0 | 0.02 |
| Deliberate | 0.43 | -0.63 | -0.19 | 0.1 |

**Table S3**: PCA scores of the multidimensional experience sampling questions in daily life

| Questions | Detail | Off-Task | Modality | Emotion |
| --- | --- | --- | --- | --- |
| Task | 0.37 | -0.42 | -0.04 | -0.30 |
| Future | 0.11 | 0.81 | 0.12 | 0.15 |
| Past | 0.06 | -0.02 | -0.07 | 0.77 |
| Self | -0.02 | 0.75 | 0.03 | 0.05 |
| Person | 0.09 | 0.17 | 0.22 | 0.57 |
| Emotion | 0.19 | 0.04 | 0.45 | -0.33 |
| Images | 0.18 | 0.05 | 0.83 | 0.23 |
| Words | 0.35 | 0.02 | -0.75 | -0.04 |
| Vivid | 0.64 | -0.05 | 0.32 | 0.30 |
| Detailed | 0.80 | -0.02 | -0.02 | 0.10 |
| Habit | 0.30 | 0.46 | -0.20 | 0.21 |
| Evolving | 0.69 | 0.18 | -0.5 | -0.09 |
| Deliberate | 0.51 | 0.08 | -0.01 | -0.46 |

Table S4: Mean, SD for individual MDSE question in Laboratory, Daily Life and difference between the two contexts

| **Scores: 0-1** | **Laboratory** | **Daily Life** | **Difference (Lab – DL)** |
| --- | --- | --- | --- |
| Task | M = .5684, SD = .2868 | M = .6849, SD = .2872 | M = -.0694, SD = .1719 |
| Future | M = .4771, SD = .3062 | M = .5725, SD = .2663 | M = -.0630, SD = .1740 |
| Past | M = .4090, SD = .2923 | M = .3726, SD = .2201 | M = .0702, SD = .1589 |
| Self | M = .5314, SD = .3048 | M = .6479, SD = .2549 | M = -.0859, SD = .1721 |
| Person | M = .4203, SD = .3189 | M = .4141, SD = .1788 | M = .0347, SD = .1495 |
| Emotion | M = .5670, SD = .1873 | M = .8813, SD = .2930 | M = -.2790, SD = .1413 |
| Images | M = .5290, SD = .3009 | M = .5236, SD = .2387 | M = .0330, SD = .1713 |
| Words | M = .6128, SD = .2949 | M = .6095, SD = .2459 | M = .0485, SD = .1793 |
| Vivid | M = .4944, SD = .2748 | M = .5281, SD = .2098 | M = -.0249, SD = .1358 |
| Detailed | M = .5570, SD = .2694 | M = .5888, SD = .2096 | M = .0057, SD = .1693 |
| Habit | M = .5778, SD = .2611 | M = .5356, SD = .2288 | M = .0568, SD = .1429 |
| Evolving | M = .4747, SD = .2733 | M = .5276, SD = .2191 | M = -.0097, SD = .1796 |
| Deliberate | M = .5326, SD = .2963 | M = .8154, SD = .3487 | M = -.2435, SD = .2074 |

Fig S1. Sample set of stimuli for the localizer fMRI task

Faces


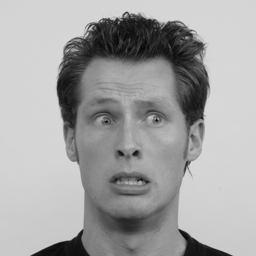

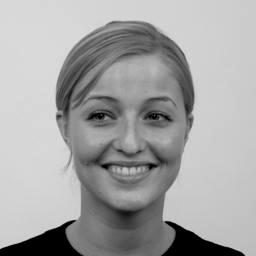

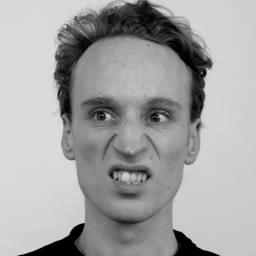

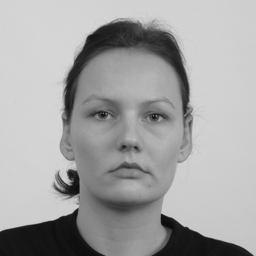

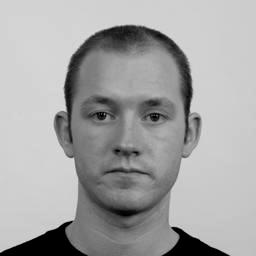

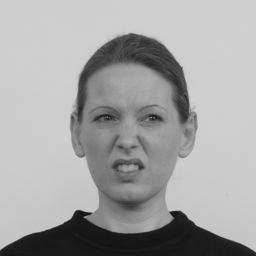


Scenes


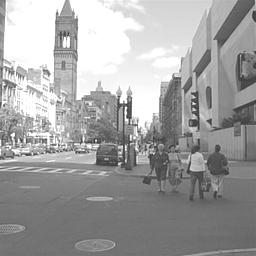

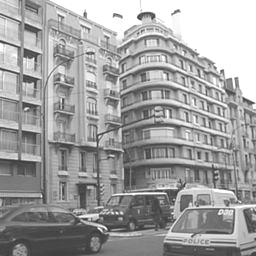

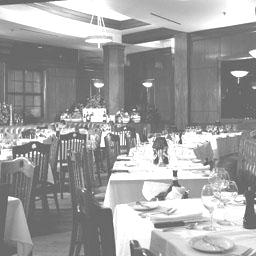

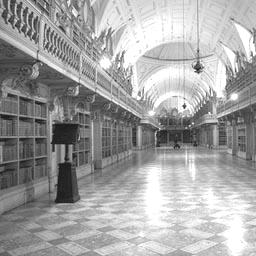

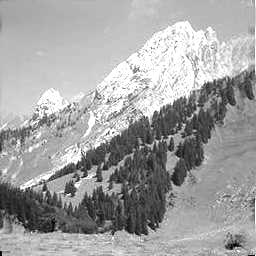

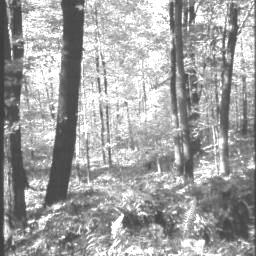


Fig S 2. Scree plots showing the decomposition of experience sampling data in the laboratory, in daily life and by combining results from both contexts. Four main Principal components were extracted from each analysis based on components having eigenvalues above 1 and a point of inflexion suggesting a large amount of variance is explained by that component.


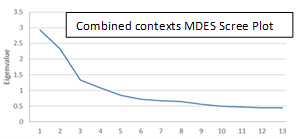


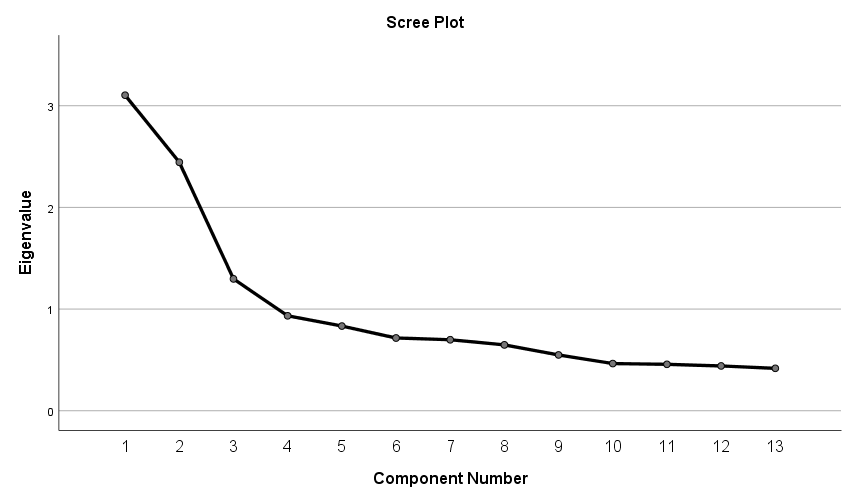

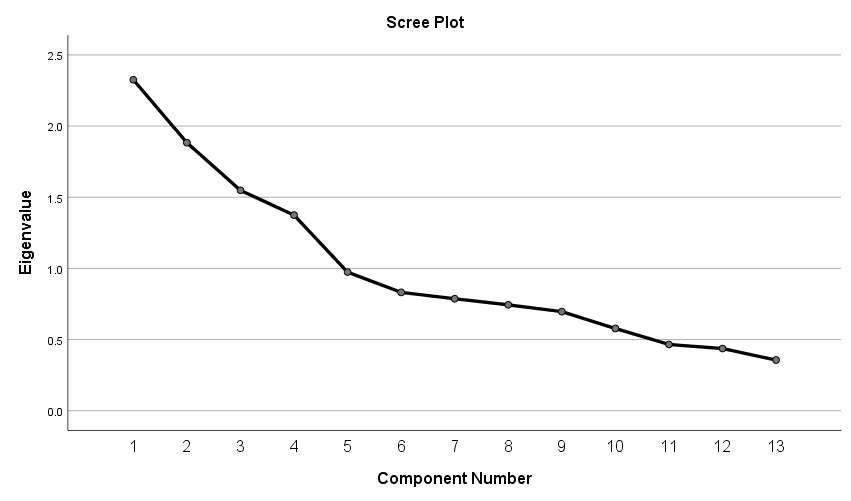


Daily Life MDES Scree Plot

Laboratory MDES Scree Plot
